# Analysis of clinicopathological features and oncological outcomes of ulcerative colitis-associated colorectal cancer based on macroscopic classification

**DOI:** 10.1007/s00384-025-05001-w

**Published:** 2025-10-29

**Authors:** Ayano Saito, Yuichiro Yokoyama, Motoi Uchino, Hiroki Ikeuchi, Koji Okabayashi, Shiro Oka, Daijiro Higashi, Shimpei Ogawa, Kazuhiro Watanabe, Masatsune Shibutani, Yoshiki Okita, Toshifumi Wakai, Yusuke Mizuuchi, Kinya Okamoto, Kazutaka Yamada, Yu Sato, Takayuki Ogino, Hideaki Kimura, Kenichi Takahashi, Koya Hida, Yusuke Kinugasa, Fumio Ishida, Junji Okuda, Koji Daito, Takayuki Yamamoto, Seiichiro Yamamoto, Fumikazu Koyama, Tsunekazu Hanai, Koji Komori, Dai Shida, Junya Arakaki, Fumihiko Fujita, Shigeki Yamaguchi, Hideki Ueno, Keiji Matsuda, Atsuo Maemoto, Riichiro Nezu, Shin Sasaki, Eiji Sunami, Tatsuki Noguchi, Kenichi Sugihara, Yoichi Ajioka, Soichiro Ishihara, Study Group for Inflammatory Bowel Disease Associated Intestinal Cancers by the Japanese Society for Cancer of the Colon, Rectum

**Affiliations:** 1 Department of Surgical Oncology, The University of Tokyo, Tokyo, Japan; 2 Department of Inflammatory Bowel Disease Surgery, Hyogo Medical University, Nishinomiya, Japan; 3 Department of Surgery, Keio University School of Medicine, Tokyo, Japan; 4 Department of Gastroenterology, Graduate School of Biomedical and Health Sciences, Hiroshima University, Hiroshima, Japan; 5 Department of Surgery, Fukuoka University Chikushi Hospital, Chikushino, Japan; 6 Department of Surgery, Division of Inflammatory Bowel Disease Surgery, Tokyo Women’s Medical University, Tokyo, Japan; 7 Department of Surgery, Tohoku University Graduate School of Medicine, Sendai, Japan; 8 Department of Gastroenterological Surgery, Osaka Metropolitan University Graduate School of Medicine, Osaka, Japan; 9 Department of Gastrointestinal and Pediatric Surgery, Institute of Life Sciences, Mie University Graduate School of Medicine, Tsu, Japan; 10 Division of Digestive and General Surgery, Graduate School of Medical and Dental Sciences, Niigata University, Niigata, Japan; 11 Department of Surgery and Oncology, Graduate School of Medical Sciences, Kyushu University, Fukuoka, Japan; 12 Department of Coloproctology, Tokyo Yamate Medical Center, Tokyo, Japan; 13 Department of Surgery, Coloproctology Center, Takano Hospital, Kumamoto, Japan; 14 Department of Surgery, Toho University Sakura Medical Center, Chiba, Japan; 15 Department of Gastroenterological Surgery, Graduate School of Medical, Osaka University, Osaka, Japan; 16 Inflammatory Bowel Disease Center, Yokohama City University Medical Center, Yokohama, Japan; 17 Department of Colorectal Surgery, Tohoku Rosai Hospital, Sendai, Japan; 18 Department of Surgery, Kyoto University Hospital, Kyoto, Japan; 19 Department of Gastrointestinal Surgery, Institute of Science Tokyo, Tokyo, Japan; 20 Digestive Disease Center, Showa University Northern Yokohama Hospital, Kanagawa, Japan; 21 Department of General and Gastroenterological Surgery, Osaka Medical and Pharmaceutical University, Takatsuki, Japan; 22 Department of Surgery, Faculty of Medicine, Kindai University, Osaka, Japan; 23 Inflammatory Bowel Disease Center, Yokkaichi Hazu Medical Center, Yokkaichi, Japan; 24 Department of Gastroenterological Surgery, Tokai University School of Medicine, Tokyo, Japan; 25 Department of Surgery, Nara Medical University, Nara, Japan; 26 Department of Surgery, School of Medicine, Fujita Health University, Aichi, Japan; 27 Department of Gastroenterological Surgery, Aichi Cancer Center Hospital, Aichi, Japan; 28 Department of Surgery, IMSUT Hospital, The Institute of Medical Science, The University of Tokyo, Tokyo, Japan; 29 Center for Gastroenterology, Department of Surgery, Urasoe General Hospital, Okinawa, Japan; 30 Department of Surgery, Kurume University Hospital, Kurume, Japan; 31 Department of Gastroenterological Surgery, Saitama Medical University International Medical Center, Saitama, Japan; 32 Department of Surgery, National Defense Medical College, Tokorozawa, Japan; 33 Department of Surgery, Teikyo University School of Medicine, Tokyo, Japan; 34 Inflammatory Bowel Disease Center, Sapporo-Higashi Tokushukai Hospital, Sapporo, Japan; 35 Department of Surgery, Nishinomiya Municipal Central Hospital, Nishinomiya, Japan; 36 Department of Coloproctological Surgery, Japanese Red Cross Medical Center, Tokyo, Japan; 37 Department of Surgery, Kyorin University, Tokyo, Japan; 38 Institute of Science Tokyo, Tokyo, Japan; 39 Department of Pathology, Niigata University, Niigata, Japan

**Keywords:** Ulcerative colitis, Ulcerative colitis associated cancer, Macroscopic classification, Prognosis

## Abstract

**Purpose:**

Few studies have reported the association of macroscopic classification with clinicopathological characteristics and prognosis of ulcerative colitis-associated colorectal cancer (UC-CRC), unlike sporadic CRC. In this study, we aimed to clarify the clinical significance of macroscopic classification of UC-CRC.

**Methods:**

The cohort included 480 patients with UC-CRC with invasion beyond the muscularis propria treated at 43 Japanese institutions between 1983 and 2023. The patients were divided into six groups based on the macroscopic type (types 0–5), and clinicopathological features and prognoses were compared.

**Results:**

Among 480 patients, 66 (13.8%), 75 (15.6%), 116 (24.2%), 63 (13.1%), 68 (14.2%), and 92 (19.2%) had type 0–5 tumors, respectively. There were significant differences in the clinicopathological characteristics with a younger age in type 4 or 5 tumors than in type 2 tumors (p < 0.01) and a higher frequency of undifferentiated carcinomas (p < 0.01) and lymph node metastasis (p < 0.01) and more advanced depth of invasion (p < 0.01) in type 4 tumors than in type 1 or 2 tumors. Type 4 and 5 were independent risk factors for 5-year recurrence-free survival (p = 0.02; type 4 [HR: 6.35], type 5 [HR: 5.25]) and type 0, 4, and 5 for overall survival (p = 0.02; type 0 [HR: 4.51], type 4 [HR: 5.70], type 5 [HR: 4.02]).

**Conclusions:**

Type 0, 4, and 5 tumors were characteristic macroscopic types of UC-CRC and correlated with worse prognosis. Therefore, endoscopic diagnosis of the macroscopic type of UC-CRC might be helpful in determining tumor aggressiveness.

## Introduction

There is an increasing trend in the number of patients with ulcerative colitis (UC), [1, 2] and those with longstanding UC are at a higher risk of developing colorectal cancer (CRC). [3] The prevalence of CRC among patients with UC is 8% at 20 years after the initial UC diagnosis and increases to 18% at 30 years. [4] With recent advancements in UC medications, the prevalence of surgical treatment for patients refractory to medications has reduced, whereas the prevalence of surgical indications for UC-associated CRC (UC-CRC) has increased owing to the increase in the number of longstanding UC cases. [5] With the increasing incidence of UC-CRC, it is crucial to understand its clinicopathological features.

UC-CRC has clinicopathological features that are different from those of sporadic CRC. [6, 7] When adjusted for the stage at diagnosis, UC-CRC has a worse prognosis than sporadic CRC. Pathologically, the prevalence of the undifferentiated types, such as mucinous or signet ring cell carcinomas, is higher among UC-CRC cases than among sporadic CRC cases, [8] and morphologically, the proportions of superficial- and invasive-type lesions are also higher in UC-CRC cases than in sporadic CRC cases. [6] UC-CRC and sporadic CRC have different carcinogenic pathways, and these different genetic mutation backgrounds may influence the difference in their clinicopathological characteristics. [9–12].

**Fig. 1:**
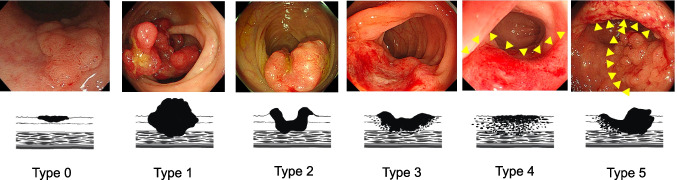
Macroscopic types defined in the Japanese Society for Cancer of the Colon and Rectum (JSCCR). The yellow arrow outlines the tumor margin

In Japan, macroscopic classification of CRC is routinely determined by colonoscopy. According to the Japanese Society for Cancer of the Colon and Rectum (JSCCR), [13] the main macroscopic types are superficial (type 0), polypoid (type 1), ulcerated with clear margin (type 2), ulcerated with infiltration (type 3), diffusely infiltrating (type 4), and unclassified (type 5) (Fig. 1). Lesions presume to be Tis and T1 cancers by colonoscopy are classified as type 0, and tumors considered to invade beyond the muscularis propria by colonoscopy are classified as Types 1–5.

The macroscopic features of tumors are readily determined using colonoscopy and can be influenced by background genetic features. The frequency of mutations in several genes, such as Kirsten rat sarcoma viral oncogene homolog (*K-Ras*), tumor protein p53 (*TP53*), and phosphatidylinositol-4,5-bisphosphate 3-kinase catalytic subunit alpha (*PIK3CA*), has been reported to vary depending on the macroscopic type of CRC. [14, 15] Therefore, macroscopic classification is considered helpful in understanding the underlying carcinogenesis of CRC, which can influence the clinicopathological features of CRC.

Additionally, macroscopic classification is reported to be an independent risk factor for recurrence of sporadic CRC and is associated with poor survival in patients; however, few studies exist on the association of macroscopic classification with clinicopathological characteristics and prognosis of UC-CRC. [16, 17] Thus, in this study, we aimed to clarify the differences in clinicopathological characteristics and oncological prognoses among the macroscopic types of UC-CRC using a large multicenter cohort.

## Method

### Patients

**Fig. 2:**
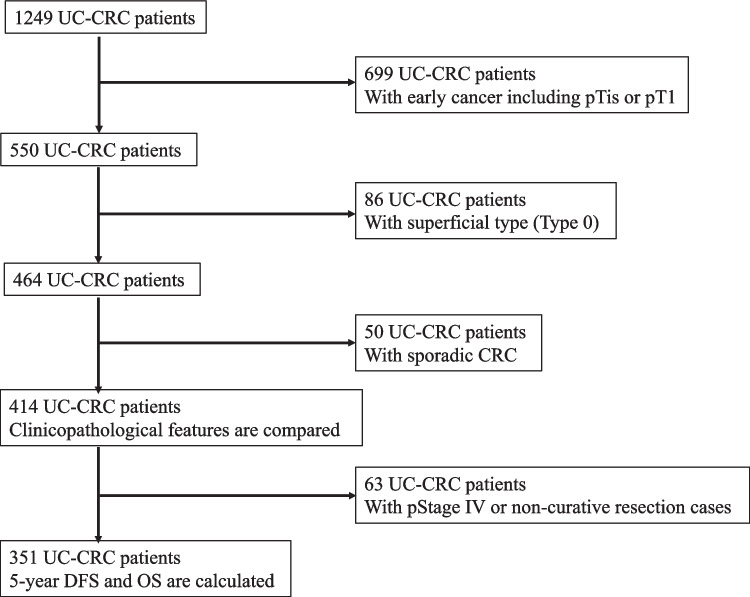
Flow diagram of patient inclusion

This study was reported in accordance with the STROBE guidelines. The medical records of patients with UC diagnosed with CRC between 1983 and 2023 were retrospectively collected from 43 institutions, including surgery and gastroenterology departments, within the JSCCR. The data were sent to the Department of Surgical Oncology at the University of Tokyo for further analysis. Cancer stage was determined according to the Tumor-Node-Metastasis Classification of Malignant Tumors, 8th Edition, of the Union for International Cancer. [18] Among 1249 patients with UC-CRC, those with early cancer, including pTis (pathologically, the tumor is confined to the mucosa and does not invade the submucosa) or pT1 (pathologically, the tumor is confined to the SM and does not invade the muscularis propria) [13] cancer, unclear macroscopic type, or sporadic CRC were excluded. Therefore, 480 patients with UC-CRC were retrospectively analyzed (Fig. 2). Baseline information on patient characteristics, treatment details, histopathological findings, and long-term oncological outcomes was collected. The patients were divided into six groups based on macroscopic types defined in the Japanese classification [13, 19]: Types 0–5. In patients with multiple lesions, only the most advanced lesions were analyzed. Factors compared among the six groups were: sex, age at UC diagnosis, disease duration, the extent of inflammation, age at UC-CRC diagnosis, the primary site of the main tumor, depth of the main tumor, lymph node metastasis, pathological stage (pStage), lymphatic or venous invasion, histology, and concurrent dysplasia. Excluding pStage IV and non-curative resection cases, 5-year recurrence-free survival (RFS) and overall survival (OS) rates were also compared.

### Statistical analysis

Statistical analyses were performed using JMP Pro 17 (SAS Institute Inc., Cary, NC, USA). Categorical variables were compared using Pearson’s chi-square test, whereas continuous variables were categorized into six groups using analysis of variance. Survival analysis was performed using the Kaplan–Meier method and compared using the log-rank test. Variables with p < 0.1 in univariate analyses were subjected to multivariate Cox proportional hazards analyses to generate hazard ratios (HRs) with 95% confidence intervals (CIs). Statistical significance was set at p < 0.05. Owing to the retrospective registration of patient data, there were missing data in the dataset. As the percentage of missing data for most variables was < 10%, we excluded missing data for each analysis in this study.

## Results

### Clinical characteristics

Patients’ clinicopathological features are presented in Table 1. Among the 480 patients, 13.8%, 15.6%, 24.2%, 13.1%, 14.2%, and 19.2% had type 0, 1, 2, 3, 4, and 5 tumors, respectively. Patients with type 0, 3, 4 or 5 were significantly younger than those with type 2 (type 0: p = 0.01, type 3, 4, 5: p < 0.01). Similarly, the median ages at CRC diagnosis of patients with type 0, 1, 3, 4, or 5 was also significantly younger than those with type 2 (type 0, 1: p = 0.03, type 3, 4, 5: p < 0.01). No significant differences in sex, duration of UC, extent of inflammation, and the primary site of the main tumor were observed among the tumor types.

**Table 1:** Clinicopathological features of patients with UC-CRC according to the macroscopic classification of tumors

Variable		Type 0 (n = 66)	Type 1 (n = 75)	Type 2 (n = 116)	Type 3 (n = 63)	Type 4 (n = 68)	Type 5 (n = 92)	p
Age at diagnosis of UC, years	Median (IQR)	32.9 (22–45)	35.9 (23–48)	40.7 (25–53)	30.7 (20–40)	32.2 (21–42)	30.5 (20–38)	< 0.01
Sex, n (%)	Male	42 (64.6%)	48 (64.0%)	71 (61.7%)	38 (60.3%)	47 (69.1%)	54 (58.7%)	0.82
	Female	23 (35.4%)	27 (36.0%)	44 (38.3%)	25 (39.7%)	21 (30.9%)	38 (41.3%)	
Duration of UC, years	Median (IQR)	19.3 (13–23)	16.2 (8–21)	16.8 (10–25)	19.9 (13–26)	16.7 (11–23)	18.4 (12–25)	0.22
Extent of inflammation, n (%)	Total colitis	54 (87.1%)	56 (75.7%)	86 (74.8%)	51 (82.3%)	54 (81.8%)	75 (84.3%)	0.29
	Left-sided colitis	8 (12.9%)	18 (24.3%)	29 (25.2%)	11 (17.7%)	12 (18.2%)	14 (15.7%)	
Age at diagnosis of CRC, years	Median (IQR)	51.6 (42–61)	51.8 (44–61)	57.6 (48–68)	50.0 (38–59)	48.8 (37–59)	48.9 (41–58)	< 0.01
Primary site of the main tumor, n (%)	right-side colon	15 (22.7%)	14 (18.7%)	31 (26.7%)	11 (17.5%)	8 (11.8%)	16 (17.8%)	0.20
	left-side colon	51 (77.3%)	61 (81.3%)	85 (73.3%)	52 (82.5%)	60 (88.2%)	74 (82.2%)	
Depth of the main tumor, n (%)	pT2	37 (56.1%)	35 (46.7%)	19 (16.4%)	3(4.8%)	4 (5.9%)	20 (21.7%)	< 0.01
	pT3	27 (40.9%)	36 (48.0%)	78 (67.2%)	33 (52.4%)	29 (42.7%)	46 (50.0%)	
	pT4a/T4b	2 (3.0%)	4 (5.3%)	19 (16.4%)	27 (42.9%)	35 (51.5%)	26 (28.3%)	
Lymph node metastasis, n (%)	Absent	53 (84.1%)	55 (73.3%)	63 (55.3%)	27 (43.6%)	22 (33.3%)	47 (52.8%)	< 0.01
	Present	10 (15.9%)	20 (26.7%)	51 (44.7%)	35 (45.4%)	44 (66.7%)	42 (47.2%)	
pStage of the main tumor, n (%)	pStageI	31 (49.2%)	30 (40.0%)	16 (13.9%)	3 (4.8%)	4 (5.9%)	13 (14.4%)	< 0.01
	pStageII	22 (34.9%)	25 (33.3%)	46 (40.0%)	23 (37.1%)	16 (23.5%)	31 (34.4%)	
	pStageIII	10 (15.9%)	20 (26.7%)	46 (40.0%)	27 (43.6%)	27 (39.7%)	38 (42.2%)	
	pStageIV	0 (0%)	0 (0%)	7 (6.1%)	9 (14.5%)	21 (30.9%)	8 (8.9%)	
Histology, n (%)	Differentiated	51 (81.0%)	58 (79.5%)	86 (76.1%)	38 (62.3%)	36 (53.7%)	57 (65.5%)	< 0.01
	Undifferentiated	12 (19.0%)	15 (20.5%)	27 (23.9%)	23 (37.7%)	31 (46.3%)	30 (34.5%)	
Lymphatic invasion, n (%)	Absent	36 (54.6%)	36 (48.0%)	46 (39.7%)	16 (25.4%)	13 (19.2%)	30 (32.6%)	< 0.01
	Present	30 (45.4%)	38 (50.7%)	69 (59.5%)	45 (71.4%)	51 (75.0%)	58 (63.0%)	
Venous invasion, n (%)	Absent	43 (65.2%)	38 (50.7%)	53 (45.7%)	16 (25.4%)	16 (23.5%)	35 (38.0%)	< 0.01
	Present	22 (33.3%)	35 (46.7%)	62 (53.5%)	45 (71.4%)	48 (70.6%)	53 (57.6%)	
Concurrent dysplasia, n (%)	Absent	19 (29.2%)	31 (41.9%)	52 (46.0%)	27 (45.8%)	26 (39.4%)	35 (38.9%)	0.23
	Present	42 (64.6%)	36 (48.7%)	49 (43.4%)	23 (39.0%)	30 (45.5%)	47 (52.2%)	

Abbreviations: UC, ulcerative colitis; IQR, interquartile range; CRC, colorectal cancer

### Pathological characteristics

As shown in Table 1, the pathological factors significantly differed among the macroscopic types. The depth of tumor invasion was more advanced in type 3 tumors than in type 0, 1, 2, in type 4 tumors compared to type 0, 1, 2, or 5 tumors, and in type 5 tumors compared to type 0 or 1 tumors (p < 0.01). In addition, the frequency of lymph node metastases was higher in patients with type 2 or 5 tumors than in those with type 0 (p < 0.01), in patients with type 3 tumors than in those with type 0 or 1 tumors (p < 0.01), and in patients with type 4 tumors than in those with type 0, 1 (p < 0.01) or 2 tumors (p = 0.03). As a result, the prevalence of pStage significantly differed among the groups (p < 0.01). A higher proportion of undifferentiated carcinomas was observed in patients with type 4 tumors than in those with type 0, 1, or 2 tumors. In addition, the rates of lymphatic and venous invasion were higher in patients with type 3 tumors than in those with type 0 and in patients with type 4 tumors than in those with type 0 or 1 (p < 0.01).

### Prognosis

The 5-year RFS and OS rates were calculated for curatively treated non-metastatic cases, excluding pStage IV and non-curative resection cases (Fig. 2). The baseline characteristics of the 415 patients included in this cohort are presented in Table 2.

**Table 2:** Baseline characteristics of patients with UC-associated colorectal cancer excluding pStageⅣ and non-curative resection cases

Variable		Patients (n = 415)
Age at diagnosis of UC, n (%)	< 59 years	375 (90.4%)
	≥ 60 years	36 (8.7%)
Sex, n (%)	Male	260 (63.0%)
	Female	153 (37.0%)
Duration of UC, n (%)	< 10 years	80 (19.3%)
	≥ 10 years	333 (80.2%)
Extent of inflammation, n (%)	Total colitis	326 (78.6%)
	Left-sided colitis	78 (18.8%)
Age at diagnosis for CRC, n (%)	< 59 years	283 (68.2%)
	≥ 60 years	132 (31.8%)
Primary site of the main tumor, n (%)	right-side colon	86 (20.7%)
	left-side colon	327 (78.8%)
Macroscopic classification, n (%)	Type 0	64 (15.4%)
	Type 1	71 (17.1%)
	Type 2	107 (25.8%)
	Type 3	49 (11.8%)
	Type 4	45 (10.8%)
	Type 5	79 (19.0%)
pT Stage of the main tumor, n (%)	pT2/3	343 (82.7%)
	pT4a/T4b	72 (17.3%)
Lymphatic or vascular invasion	Absent	122 (29.4%)
	Present	285 (68.7%)
Lymph node metastasis, n (%)	Absent	252 (60.7%)
	Present	158 (38.1%)
Histology, n (%)	Differentiated	302 (72.8%)
	Undifferentiated	99 (23.9%)
Concurrent dysplasia, n (%)	Absent	165 (40.8%)
	Present	204 (50.5%)

Abbreviation: UC, ulcerative colitis; CRC, colorectal cancer

**Fig. 3:**
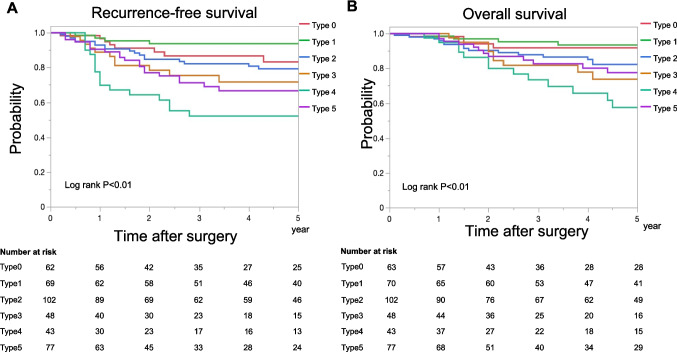
Recurrence-free survival and overall survival according to macroscopic type. Recurrence-free survival (A) and overall survival (B). Red, type 0; light green, type 1; blue, type 2; brown, type 3; green, type 4; purple, type 5

The 5-year RFS and OS rates significantly differed among the macroscopic types (Fig. 3A and B). The 5-year RFS rates for patients with type 0, 1, 2, 3, 4, and 5 tumors were 83.2%, 93.7%, 78.4%, 74.6%, 49.7%, and 66.6%, respectively. The 5-year OS rates for patients with type 0, 1, 2, 3, 4, and 5 tumors were 92.0%, 93.4%, 82.2%, 73.7%, 57.5%, and 78.0%, respectively.

**Fig. 4:**
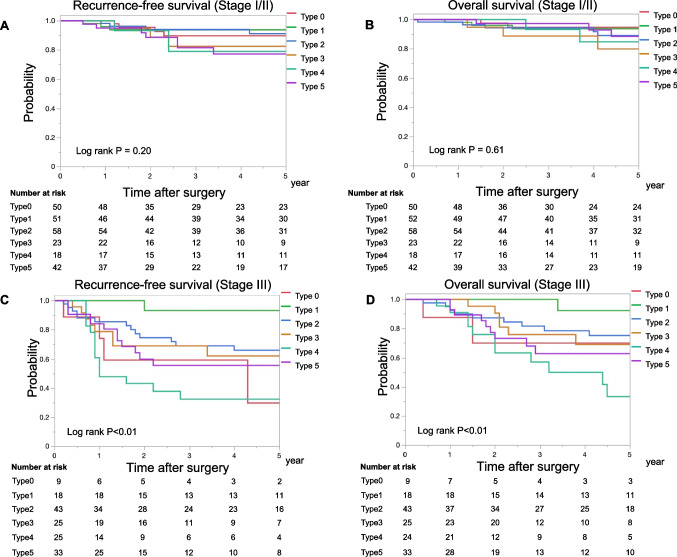
Recurrence-free survival and overall survival according to macroscopic type adjusted for pathological stages Recurrence-free survival (A) and overall survival (B) in stage I/II UC-CRC, and recurrence-free survival (C) and overall survival (D) in stage III UC-CRC. Red, type 0; light green, type 1; blue, type 2; brown, type 3; green, type 4; purple, type 5

When stratified by pathological stage, in pStage I/II cancers, 5-year RFS rates for patients with type 0, 1, 2, 3, 4, and 5 were 89.8%, 93.8%, 91.2%, 82.5%, 79.0%, and 77.3%, respectively, which was not significantly different among the groups (Fig. 4A). Likewise, the 5-year OS rates were 94.5%, 93.8%, 89.2%, 79.9%, 84.9%, and 88.6%, respectively, and were not significantly different among the groups (Fig. 4B). In pStage III cancers, the 5-year RFS rates (Fig. 4C) for patients with type 0, 1, 2, 3, 4, and 5 were 29.6%, 93.3%, 65.9%, 59.5%, 32.3%, and 55.6%, respectively and 5-year OS rates (Fig. 4D) were 70.0%, 92.3%, 75.3%, 69.0%, 33.2%, and 62.8%, respectively, which were significantly different among the groups (p < 0.01).

Subsequently, multivariate Cox proportional hazards analyses were performed for the significant factors related to RFS and OS. Regarding the RFS, differences in duration of UC (p = 0.07), extent of inflammation (p = 0.09), macroscopic features (p < 0.01), depth of the main tumor (p < 0.01), lymphatic or vascular invasion (p < 0.01), lymph node metastasis (p < 0.01), histology (p < 0.01), and concurrent dysplasia (p = 0.07) were observed in univariate analyses (Table 3). Multivariate Cox proportional hazards analyses revealed that pT4 (p = 0.02; HR: 2.22, 95% CI: 1.21–4.07]), lymph node metastasis (p < 0.01; HR: 3.65, 95% CI: 2.03–6.76), macroscopic classification (p < 0.01; type 4 HR: 6.35, 95% CI: 1.69–23.8, and type 5 HR: 5.25, 95% CI: 1.46–18.9) were independent prognostic factors for RFS (Table 3). Regarding the OS, significant differences in sex (p = 0.02), duration of UC (p = 0.02), macroscopic features (p < 0.01), depth of the main tumor (p < 0.01), lymphatic or vascular invasion (p < 0.01), lymph node metastasis (p < 0.01), and histology (p < 0.01) were observed in univariate analyses (Table 4). Multivariate Cox proportional hazards analyses revealed that lymphatic or vascular invasion (p = 0.04; HR: 2.14, 95% CI: 1.02–5.10) and lymph node metastasis (p < 0.01; HR: 2.94, 95% CI: 1.64–5.38) were independent prognostic factors for OS. Moreover, the macroscopic classifications type 0 (HR: 4.51, 95% CI: 1.20–16.9), type 4 (HR, 5.70; 95% CI, 1.81–18.0), and type 5 (HR: 4.02, 95% CI: 1.28–12.7) were independent prognostic factors for OS (p = 0.02; Table 4).

**Table 3:** Univariate and multivariate analyses of risk factors for RFS

		Univariate		Multivariate		
		5-year RFS (%)	*p*-Value	HR	95% CI	*p*-Value
Age at diagnosis of UC	< 59 years	76.0	0.20			
	≥ 60 years	84.9				
Sex	Male	74.5	0.38			
	Female	79.0				
Duration of UC	< 10 years	66.2	0.07	1		0.29
	≥ 10 years	79.1		0.71	0.39–1.36	
Extent of inflammation	Total colitis	77.9	0.09	1		0.22
	Left-sided colitis	68.7		1.55	0.76–2.91	
Age at diagnosis of CRC	< 59 years	73.7	0.11			
	≥ 60 years	81.8				
Primary site of the main tumor	right-side colon	80.7	0.22			
	left-side colon	75.0				
Macroscopic classification			< 0.01			0.02
	Type 0	83.2		4.24	0.96–18.8	
	Type 1	93.7		1		
	Type 2	79.3		2.76	0.78–9.81	
	Type 3	70.1		2.28	0.54–9.59	
	Type 4	52.0		6.35	1.69–23.8	
	Type 5	66.6		5.25	1.46–18.9	
pT Stage of the main tumor	pT2/3	82.2	< 0.01	1		0.02
	pT4a/T4b	48.6		2.22	1.21–4.07	
Lymphatic or vascular invasion	Absent	88.2	< 0.01	1		0.12
	Present	71.2		1.81	0.86–4.29	
Lymph node metastasis, n (%)	Absent	87.2	< 0.01	1		< 0.01
	Present	58.5		3.65	2.03–6.76	
Histology, n (%)	Differentiated	81.0	< 0.01	1		0.61
	Undifferentiated	64.9		1.16	0.64–2.06	
Concurrent dysplasia, n (%)	Absent	73.5	0.07	1		0.18
	Present	81.6		1.21	0.42–1.19	

Abbreviations: UC, ulcerative colitis; CRC, colorectal cancer; RFS, recurrence-free survival; CI, confidence interval; HR, hazard ratio

**Table 4:** Univariate and multivariate analyses of risk factors for OS

		Univariate			Multivariate		
		5-year OS (%)	*p*-Value		HR	95% CI	*p*-Value
Age at diagnosis of UC	< 59 years	77.5	0.89				
	≥ 60 years	82.2					
Sex	Male	77.4	0.02		1		0.03
	Female	88.4			0.54	0.28–0.96	
Duration of UC	< 10 years	69.9	0.02		1		0.08
	≥ 10 years	84.6			0.56	0.31–1.08	
Extent of inflammation	Total colitis	82.4	0.35				
	Left-sided colitis	76.3					
Age at diagnosis of CRC	< 59 years	78.9	0.41				
	≥ 60 years	87.0					
Primary site of the main tumor	right-side colon	90.0	0.13				
	left-side colon	79.0					
Macroscopic classification			<0.01				0.02
	Type 0	92.0			4.51	1.20–16.9	
	Type 1	93.4			1		
	Type 2	82.2			2.23	0.72–6.87	
	Type 3	73.7			2.56	0.72–9.06	
	Type 4	57.5			5.70	1.81–18.0	
	Type 5	78.0			4.02	1.28–12.7	
pT Stage of the main tumor	pT2/3	85.4	<0.01		1		0.13
	pT4a/T4b	62.4			1.63	0.86–3.00	
Lymphatic or vascular invasion	Absent	93.7	<0.01		1		0.04
	Present	75.7			2.14	1.02–5.10	
Lymph node metastasis, n (%)	Absent	90.2	<0.01		1		<0.01
	Present	67.0			2.94	1.64–5.38	
Histology, n (%)	Differentiated	86.6	<0.01		1		0.27
	Undifferentiated	69.5			1.38	0.77–2.41	
Concurrent dysplasia, n (%)	Absent	81.6	0.74				
	Present	81.5					

Abbreviations: UC, ulcerative colitis; CRC, colorectal cancer; OS, overall survival; CI, confidence interval; HR, hazard ratio

## Discussion

In the present study, there was a close association between the clinicopathological characteristics and macroscopic classification of UC-CRC. Moreover, we found that macroscopic features correlated with oncological outcomes, such as RFS and OS.

First, we evaluated the distribution of the macroscopic patterns of UC-CRC. In a previous report, among sporadic CRC cases invading deeper than the submucosa, type 2 tumors were dominant and accounted for 68% of CRC cases, followed by type 3 (12%), type 1 (10%), and type 0 (8%) tumors, whereas type 4 and 5 tumors were scarce (both 1%). [6] In the present study, 13.8%, 15.6%, 24.2%, 13.1%, 14.2%, and 19.2% of patients with UC-CRC had type 0, 1, 2, 3, 4, and 5 tumors, respectively. As previously reported, UC-CRC is characterized by a lower proportion of localized ulcerative type and a higher proportion of infiltration with an ill-defined edge tumor (type 4) and non-classifiable type (type 5) than sporadic CRC. [6] Furthermore, considering that our study included only tumors developing deeper than muscularis propria and that ordinally lesions presumed to be pTis and pT1 cancers are classified as superficial (type 0), the high proportion of type 0 tumors with invasion beyond the muscularis propria is also considered to be characteristic of UC-CRC. [18] Noguchi T reported that even with annual surveillance endoscopy, 27% of patients with UC-CRC were diagnosed as having cancer with invasion beyond the muscularis propria. This is probably associated with the high prevalence of type 0 tumors with invasion beyond the muscularis propria in UC-CRC, which is difficult to detect in the early stage by surveillance endoscopy. [20]

Macroscopic features are reported to be influenced by genetic mutation patterns. The mutation frequency of *K-Ras* is significantly lower in flat adenomas compared to polypoid adenomas or CRC. [13] Other mutations of genes, such as *TP53* and *PIK3CA*, and epigenetic mutations, such as LINE-1 hypomethylation, also influence the macroscopic morphology. [14] Therefore, the morphological difference between UC-CRC and sporadic CRC may be due to the different carcinogenic pathways. Sporadic CRC arises from the accumulation of genetic mutations, known as the adenocarcinoma sequence, whereas UC-CRC arises from genetic mutations induced by chronic inflammation, known as the inflammation-dysplasia–carcinoma sequence. [9–12] This difference in carcinogenesis results in different genetic mutation patterns, [21] which can lead to different macroscopic patterns in UC-CRC and sporadic CRC.

The most important finding of the present study was determining the clinicopathological features of UC-CRC based on macroscopic features. UC-CRC has an earlier occurrence, worse outcome, and a higher rate of aggressive histological features, such as mucinous or signet ring cell type, than sporadic CRC. [6, 22] This difference may be due to differences in the genetic mutation backgrounds of UC-CRC and sporadic CRC. However, it is difficult to clearly divide CRC into UC-CRC and sporadic CRC groups based on genetic mutation signatures. This indicates that even in UC-CRC cases, there should be a tumor with carcinogenesis and genetic characteristics similar to those of sporadic CRC. In the present study, we found that patients with type 0, 1, 3, 4, and 5 tumors were younger than those with type 2 tumors. A higher proportion of undifferentiated carcinomas was also observed in patients with type 4 tumors than in those with type 0, 1, or 2 tumors. The depth of tumor invasion was more advanced in type 3 tumors than in type 0, 1, or 2 tumors, in type 4 tumors than in type 0, 1, 2, or 5 tumors, and in type 5 tumors than in type 0 or 1 tumors. In addition, the frequency of lymph node metastases was higher in patients with type 3 tumors than in those with type 0 or 1 tumors and in patients with type 4 tumors than in those with type 0, 1, or 2 tumors. As mentioned above, the tumor presumed to be early cancer endoscopically was classified as type 0 tumor, and the tumor presumed to be cancer with invasion beyond the muscularis propria was classified as type 1–5. For this reason, clinicopathological features of type 0 tumors could not be equally compared with that of the other types of tumors. Considering that localized and ulcerative types, such as type 1 and 2 tumors, are typical morphologies in sporadic CRC cases and that infiltration with an ill-defined edge tumor, particularly type 4 tumors, and non-classifiable types are characteristic morphologies in UC-CRC cases, our results indicate that, based on macroscopic classification, UC-CRC may be divided into a group with more prominent characteristics of UC-CRC and a group with similar characteristics to sporadic CRC.

Notably, our findings also revealed that macroscopic classification significantly influenced the RFS and OS of patients with UC-CRC. Several studies have revealed prognostic factors for UC-CRC, such as pStage, sex, and duration of UC. [22] In the present study, we found that 5-year RFS and OS were also significantly different among the macroscopic types. As there was a high proportion of fewer tumors with invasion beyond the muscularis propria in the type 0 tumor group, the 5-year RFS and OS were examined in a stage-matched situation. Both the 5-year RFS and OS were significantly different among macroscopic types in Stage III cases, and type 0, 4, and 5 tumors, which are characteristic macroscopic features of UC-CRC, showed poor prognosis. Multivariate Cox proportional hazards analyses revealed that, in addition to sex, duration of UC, depth of tumor invasion, lymphatic or vascular invasion, lymph node metastasis, and histology, macroscopic classification was a prognostic factor for 5-year RFS and OS in univariate analyses. In multivariate Cox proportional hazards analyses, macroscopic classification was an independent risk factor for 5-year RFS, in addition to the depth of the main tumor and lymph node metastasis, and for 5-year OS, in addition to sex, lymphatic and vascular invasion, and lymph node metastasis. Furthermore, among the macroscopic classification, type 4 and 5 tumors were significant independent risk factors for 5-year RFS and type 0, 4, and 5 for 5-year OS. Considering that patients with UC-CRC have worse survival than those with sporadic CRC, as previously reported, [6, 23, 24] these findings suggest that biological malignancy and macroscopic types might be closely associated and that type 0, 4, and 5 tumors have more prominent characteristics of UC-CRC.

This study has some limitations. First is its retrospective design, and some cases may be missing from the database of each institution, resulting in selection bias. However, collecting a large number of UC-CRC cases from a single institution is difficult, and this large-scale multicenter study of UC-CRC is a strength of our study. Second, the diagnosis of UC-CRC and sporadic CRC was established in each institution and was not centralized. These diagnoses are sometimes difficult; therefore, some sporadic CRC cases may be included as UC-CRC cases, and some UC-CRC cases may be included as sporadic CRC cases. Third, to clarify the relationship between the macroscopic type and biological malignancy, it is important to compare UC-CRC and sporadic CRC in macroscopic classification-matched situations. However, there is a large discrepancy in disease prevalence between UC-CRC and sporadic CRC; therefore, comparing these two groups in macroscopic classification-matched situations is difficult. Finally, we did not examine the differences in gene mutations among the macroscopic types of UC-CRC, which should be assessed in future studies.

In conclusion, our findings revealed that the clinicopathological features and oncological outcomes significantly differed among the macroscopic types of UC-CRC. A high proportion of type 0, 4, and 5 tumors was a significant feature of UC-CRC. Among the six macroscopic types, type 4 and type 5 tumors had clinicopathological features such as earlier occurrence and a higher rate of aggressive and histological features. Additionally, in multivariate Cox proportional hazards analyses that included factors such as sex, duration of UC, depth of tumor invasion, lymphatic or vascular invasion, lymph node metastasis and histology, type 0, 4, and 5 tumors also had worse outcomes, which had been reported to be the characteristics of UC-CRC itself. Therefore, an endoscopic diagnosis of the macroscopic classification of UC-CRC might be helpful in assessing tumor aggressiveness.

## Data Availability

No datasets were generated or analysed during the current study.
